# Aqua­(benzene-1,2-dicarboxyl­ato-κ*O*)bis­[2-(1*H*-pyrazol-3-yl-κ*N* ^2^)pyridine-κ*N*]cadmium(II)

**DOI:** 10.1107/S1600536810034616

**Published:** 2010-09-04

**Authors:** Jian-Hua Guo

**Affiliations:** a Tianjin Key Laboratory of Structure and Performance for Functional Molecules, College of Chemistry, Tianjin Normal University, Tianjin 300387, People’s Republic of China

## Abstract

In the mononuclear title complex, [Cd(C_8_H_4_O_4_)(C_8_H_7_N_3_)_2_(H_2_O)], the Cd^II^ atom is six-coordinated in a distorted octa­hedral geometry by four N atoms from two bidentate chelating 2-(1*H*-pyrazol-3-yl)pyridine ligands, one O atom from a benzene-1,2-dicarboxyl­ate ligand and one water mol­ecule. The mol­ecular structure features intra­molecular O—H⋯O and N—H⋯O hydrogen bonds. In the crystal structure, the complex mol­ecules are assembled into a two-dimensional supra­molecular layer parallel to (011) *via* O—H⋯O and N—H⋯O hydrogen bonds and π–π stacking inter­actions between the pyridyl and pyrazole rings [centroid–centroid distances = 3.544 (2) and 3.722 (3) Å].

## Related literature

For general background to the roles played by aromatic ring stacking and hydrogen bonding in biological reactions and in mol­ecular recognition and self-organization, see: Borrows *et al.* (1995 ▶); Hunter (1994 ▶). For related structures, see: Cheng *et al.* (2006 ▶); Hu *et al.* (2008 ▶); Wan *et al.* (2003 ▶).
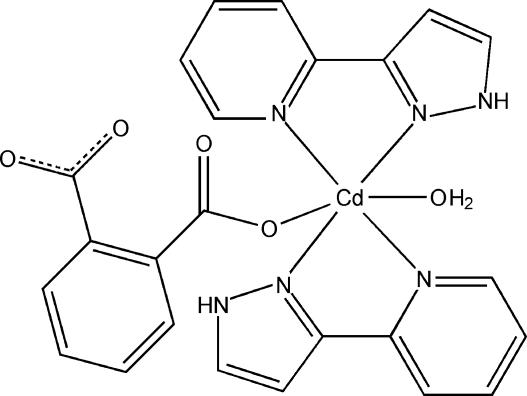


## Experimental

### 

#### Crystal data

[Cd(C_8_H_4_O_4_)(C_8_H_7_N_3_)_2_(H_2_O)]
*M* *_r_* = 584.86
Triclinic, 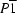

*a* = 10.1546 (7) Å
*b* = 10.7709 (7) Å
*c* = 12.4900 (9) Å
α = 64.986 (1)°
β = 71.209 (1)°
γ = 81.618 (1)°
*V* = 1171.90 (14) Å^3^
*Z* = 2
Mo *K*α radiation
μ = 0.98 mm^−1^
*T* = 296 K
0.28 × 0.22 × 0.20 mm

#### Data collection

Bruker APEXII CCD diffractometer
Absorption correction: multi-scan (*SADABS*; Sheldrick, 1996 ▶) *T*_min_ = 0.771, *T*_max_ = 0.828
5869 measured reflections
4075 independent reflections
3194 reflections with *I* > 2σ(*I*)
*R*_int_ = 0.020

#### Refinement

*R*[*F* ^2^ > 2σ(*F* ^2^)] = 0.033
*wR*(*F* ^2^) = 0.064
*S* = 1.06
4075 reflections
325 parameters
H-atom parameters constrained
Δρ_max_ = 0.63 e Å^−3^
Δρ_min_ = −0.60 e Å^−3^

### 

Data collection: *APEX2* (Bruker, 2007 ▶); cell refinement: *SAINT* (Bruker, 2007 ▶); data reduction: *SAINT*; program(s) used to solve structure: *SHELXS97* (Sheldrick, 2008 ▶); program(s) used to refine structure: *SHELXL97* (Sheldrick, 2008 ▶); molecular graphics: *SHELXTL* (Sheldrick, 2008 ▶); software used to prepare material for publication: *SHELXTL*.

## Supplementary Material

Crystal structure: contains datablocks global, I. DOI: 10.1107/S1600536810034616/hy2347sup1.cif

Structure factors: contains datablocks I. DOI: 10.1107/S1600536810034616/hy2347Isup2.hkl

Additional supplementary materials:  crystallographic information; 3D view; checkCIF report

## Figures and Tables

**Table 1:** Hydrogen-bond geometry (Å, °)

*D*—H⋯*A*	*D*—H	H⋯*A*	*D*⋯*A*	*D*—H⋯*A*
O5—H5*A*⋯O3^i^	0.85	1.84	2.672 (4)	168
O5—H5*B*⋯O3	0.85	2.07	2.892 (4)	164
N3—H3*A*⋯O4^i^	0.86	1.79	2.646 (4)	176
N6—H6⋯O2	0.86	1.91	2.700 (4)	152

Symmetry code: (i) 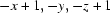.

**Fig. 1.**
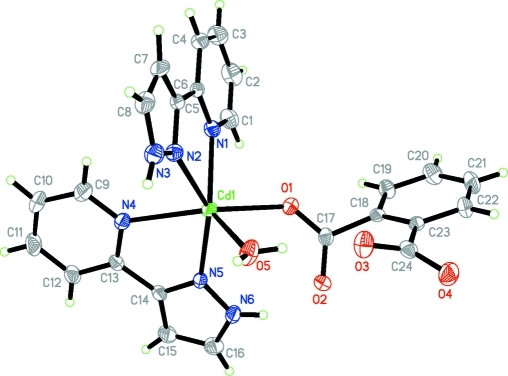
The molecular structure of the title complex, showing 30% probability displacement ellipsoids.

**Fig. 2.**
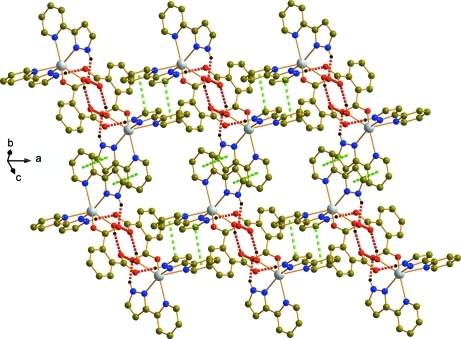
The two-dimensional supramolecular layer in the title complex, formed via O—H···O, N—H···O hydrogen bonds and π–π stacking interactions (dashed lines).

## supplementary crystallographic information

### Comment

The strategies for the construction of functional systems depend on the nature of the interactions responsible for creating networks. Aromatic ring stacking and hydrogen bonds are two important types of intermolecular non-covalent interactions, which play vital roles in highly efficient and specific biological reactions and are essential for molecular recognition and self-organization (Borrows *et al.*, 1995; Hunter, 1994). Benzene-1,2-dicarboxylic acid (1,2-H_2_bdc) exhibits rich coordination modes to metal centers owing to its rigidity and polycarboxylate groups. Therefore, its metal complexes often show two- or three-dimensional structures (Cheng *et al.*, 2006; Wan *et al.*, 2003). 3-(2-Pyridyl)pyrazole (*L*) can act as a simple bidentate chelating ligand, similar to 2,2'-bipyridine (or 1,10-phenanthroline) (Hu *et al.*, 2008). In the present paper, we report the crystal structure of the title compound, a new Cd^II^ complex based on the mixed ligands *L* and 1,2-bdc, in which interesting hydrogen-bonding and π–π stacking interactions are observed.

In the title mononuclear compound, as shown in Fig. 1, the Cd^II^ atom is coordinated by four N atoms from two *L* ligands, with Cd—N bond lengths in the range of 2.241 (3)–2.525 (3) Å, one O atom from the carboxylate group of a 1,2-bdc ligand, with a Cd—O bond distance of 2.306 (3)Å and one O atom from a water molecule, with a Cd—O bond distance of 2.306 (2) Å. Thus, the coordination polyhedron around the Cd^II^ atom can be best described as distorted octahedral. The complex molecule involves intramolecular O—H···O and N—H···O hydrogen bonds (Table 1). Analysis of the crystal packing indicates that intermolecular O—H···O and N—H···O hydrogen bonds link two complex molecules, producing a dimeric unit. The dimeric units are arranged in a parallel fashion, which makes the aromatic stacking available to afford a two-dimensional supramolecular layer (Fig. 2). The centroid–centroid distances between the pyridyl and pyrazolyl rings are 3.544 (2)Å and 3.722 (3) Å and their corresponding dihedral angles are 3.40 (2) and 4.68 (1)°, respectively.

### Experimental

The title complex was obtained by the reaction of Cd(CH_3_CO_2_)_2_.2H_2_O, 3-(2-pyridyl)pyrazole and benzene-1,2-dicarboxylic acid in a molar ratio of 1:1:1, mixed with water (10 ml) under hydrothermal conditions at 413 K for 3 d. After cooled to room temperature at a rate of 5 K h^-1^, colorless block crystals suitable for X-ray analysis were obtained in a 60% yield. Analysis, calculated for C_24_H_20_CdN_6_O_5_: C 49.29, H 3.45, N 14.37%; found: C 49.33, H 3.35, N 14.44%.

### Refinement

Although all H atoms were visible in difference Fourier maps, they were placed in geometrically calculated positions and refined as riding atoms, with C—H = 0.93, N—H = 0.86 and O—H = 0.85 Å and with *U*_iso_(H) = 1.2*U*_eq_(C, N) and *U*_iso_(H) = 1.5*U*_eq_(O).

### Crystal data

**Table:**

[Cd(C_8_H_4_O_4_)(C_8_H_7_N_3_)_2_(H_2_O)]	*Z* = 2
*M**_r_* = 584.86	*F*(000) = 588
Triclinic, *P*1	*D*_x_ = 1.657 Mg m^−^^3^
Hall symbol: -P 1	Mo *K*α radiation, λ = 0.71073 Å
*a* = 10.1546 (7) Å	Cell parameters from 2320 reflections
*b* = 10.7709 (7) Å	θ = 2.4–26.3°
*c* = 12.4900 (9) Å	µ = 0.98 mm^−^^1^
α = 64.986 (1)°	*T* = 296 K
β = 71.209 (1)°	Block, colorless
γ = 81.618 (1)°	0.28 × 0.22 × 0.20 mm
*V* = 1171.90 (14) Å^3^	

### Data collection

**Table:**

Bruker APEXII CCD diffractometer	4075 independent reflections
Radiation source: fine-focus sealed tube	3194 reflections with *I* > 2σ(*I*)
graphite	*R*_int_ = 0.020
φ and ω scans	θ_max_ = 25.0°, θ_min_ = 1.9°
Absorption correction: multi-scan (*SADABS*; Sheldrick, 1996)	*h* = −12→12
*T*_min_ = 0.771, *T*_max_ = 0.828	*k* = −11→12
5869 measured reflections	*l* = −14→12

### Refinement

**Table:**

Refinement on *F*^2^	Primary atom site location: structure-invariant direct methods
Least-squares matrix: full	Secondary atom site location: difference Fourier map
*R*[*F*^2^ > 2σ(*F*^2^)] = 0.033	Hydrogen site location: inferred from neighbouring sites
*wR*(*F*^2^) = 0.064	H-atom parameters constrained
*S* = 1.06	*w* = 1/[σ^2^(*F*_o_^2^) + (0.0213*P*)^2^ + 0.3676*P*] where *P* = (*F*_o_^2^ + 2*F*_c_^2^)/3
4075 reflections	(Δ/σ)_max_ < 0.001
325 parameters	Δρ_max_ = 0.63 e Å^−^^3^
0 restraints	Δρ_min_ = −0.60 e Å^−^^3^

### Fractional atomic coordinates and isotropic or equivalent isotropic displacement parameters (Å^2^)

**Table:**

	*x*	*y*	*z*	*U*_iso_*/*U*_eq_	
Cd1	0.24772 (3)	0.29292 (3)	0.32881 (3)	0.03231 (10)	
O1	0.1867 (3)	0.2927 (3)	0.5234 (2)	0.0476 (7)	
O2	0.3783 (3)	0.3400 (3)	0.5504 (2)	0.0436 (7)	
O3	0.3964 (3)	0.0332 (3)	0.6592 (3)	0.0526 (7)	
O4	0.4584 (3)	−0.0169 (3)	0.8281 (2)	0.0553 (8)	
O5	0.4235 (3)	0.1523 (3)	0.3978 (2)	0.0518 (7)	
H5A	0.4793	0.0992	0.3694	0.078*	
H5B	0.4031	0.1299	0.4751	0.078*	
N1	0.0044 (3)	0.2749 (3)	0.3848 (3)	0.0356 (7)	
N2	0.1955 (3)	0.1179 (3)	0.2898 (3)	0.0335 (7)	
N3	0.2667 (3)	0.0223 (3)	0.2516 (3)	0.0414 (8)	
H3A	0.3558	0.0203	0.2226	0.050*	
N4	0.2559 (3)	0.4476 (3)	0.1084 (3)	0.0391 (8)	
N5	0.3742 (3)	0.4808 (3)	0.2559 (3)	0.0337 (7)	
N6	0.4404 (3)	0.5217 (3)	0.3126 (3)	0.0428 (8)	
H6	0.4467	0.4744	0.3861	0.051*	
C1	−0.0887 (4)	0.3532 (4)	0.4319 (4)	0.0483 (11)	
H1	−0.0561	0.4242	0.4401	0.058*	
C2	−0.2294 (5)	0.3350 (5)	0.4689 (4)	0.0587 (12)	
H2	−0.2909	0.3935	0.4993	0.070*	
C3	−0.2772 (4)	0.2280 (5)	0.4598 (4)	0.0601 (13)	
H3	−0.3722	0.2125	0.4851	0.072*	
C4	−0.1854 (4)	0.1449 (4)	0.4138 (3)	0.0453 (11)	
H4	−0.2169	0.0719	0.4077	0.054*	
C5	−0.0440 (4)	0.1701 (4)	0.3760 (3)	0.0330 (9)	
C6	0.0611 (4)	0.0854 (4)	0.3267 (3)	0.0324 (9)	
C7	0.0489 (4)	−0.0336 (4)	0.3124 (3)	0.0452 (11)	
H7	−0.0324	−0.0786	0.3316	0.054*	
C8	0.1816 (5)	−0.0697 (4)	0.2642 (4)	0.0462 (11)	
H8	0.2082	−0.1450	0.2437	0.055*	
C9	0.1999 (5)	0.4256 (5)	0.0353 (4)	0.0578 (12)	
H9	0.1482	0.3466	0.0681	0.069*	
C10	0.2141 (5)	0.5130 (5)	−0.0862 (4)	0.0635 (13)	
H10	0.1728	0.4939	−0.1340	0.076*	
C11	0.2908 (4)	0.6286 (5)	−0.1340 (4)	0.0579 (12)	
H11	0.3027	0.6895	−0.2158	0.069*	
C12	0.3501 (4)	0.6549 (4)	−0.0614 (3)	0.0431 (10)	
H12	0.4024	0.7333	−0.0931	0.052*	
C13	0.3305 (3)	0.5622 (3)	0.0601 (3)	0.0312 (9)	
C14	0.3906 (4)	0.5814 (3)	0.1437 (3)	0.0313 (8)	
C15	0.4666 (4)	0.6870 (4)	0.1304 (4)	0.0485 (11)	
H15	0.4923	0.7688	0.0615	0.058*	
C16	0.4951 (4)	0.6451 (4)	0.2400 (4)	0.0529 (12)	
H16	0.5442	0.6941	0.2604	0.064*	
C17	0.2603 (4)	0.2903 (3)	0.5888 (3)	0.0330 (9)	
C18	0.1943 (3)	0.2270 (3)	0.7271 (3)	0.0284 (8)	
C19	0.0726 (4)	0.2855 (4)	0.7768 (3)	0.0423 (10)	
H19	0.0285	0.3548	0.7243	0.051*	
C20	0.0159 (4)	0.2427 (4)	0.9027 (4)	0.0514 (11)	
H20	−0.0655	0.2833	0.9347	0.062*	
C21	0.0796 (4)	0.1403 (4)	0.9804 (4)	0.0523 (11)	
H21	0.0423	0.1121	1.0655	0.063*	
C22	0.1987 (4)	0.0793 (4)	0.9329 (3)	0.0434 (10)	
H22	0.2414	0.0099	0.9866	0.052*	
C23	0.2569 (3)	0.1189 (3)	0.8060 (3)	0.0288 (8)	
C24	0.3806 (4)	0.0414 (4)	0.7593 (4)	0.0365 (9)	

### Atomic displacement parameters (Å^2^)

**Table:**

	*U*^11^	*U*^22^	*U*^33^	*U*^12^	*U*^13^	*U*^23^
Cd1	0.03555 (16)	0.02947 (15)	0.03196 (15)	−0.00863 (11)	−0.01207 (12)	−0.00811 (11)
O1	0.0392 (16)	0.0718 (19)	0.0339 (15)	−0.0109 (14)	−0.0121 (13)	−0.0188 (14)
O2	0.0391 (15)	0.0525 (17)	0.0334 (14)	−0.0165 (13)	−0.0063 (12)	−0.0098 (13)
O3	0.0638 (19)	0.0536 (18)	0.0454 (17)	0.0198 (14)	−0.0187 (15)	−0.0289 (15)
O4	0.0462 (17)	0.068 (2)	0.0481 (17)	0.0108 (15)	−0.0218 (15)	−0.0174 (15)
O5	0.0597 (18)	0.0574 (18)	0.0525 (17)	0.0234 (14)	−0.0305 (15)	−0.0327 (15)
N1	0.0378 (18)	0.0329 (18)	0.0318 (17)	−0.0021 (15)	−0.0107 (15)	−0.0080 (15)
N2	0.0373 (18)	0.0305 (17)	0.0340 (18)	−0.0020 (15)	−0.0107 (15)	−0.0134 (15)
N3	0.043 (2)	0.042 (2)	0.041 (2)	0.0027 (16)	−0.0156 (17)	−0.0165 (17)
N4	0.0442 (19)	0.0408 (19)	0.0325 (18)	−0.0096 (16)	−0.0151 (16)	−0.0092 (15)
N5	0.0397 (18)	0.0361 (17)	0.0258 (16)	−0.0100 (14)	−0.0106 (14)	−0.0089 (15)
N6	0.053 (2)	0.045 (2)	0.0325 (18)	−0.0139 (17)	−0.0164 (16)	−0.0110 (16)
C1	0.057 (3)	0.036 (2)	0.043 (2)	0.001 (2)	−0.012 (2)	−0.010 (2)
C2	0.050 (3)	0.058 (3)	0.054 (3)	0.016 (2)	−0.008 (2)	−0.020 (2)
C3	0.032 (2)	0.083 (4)	0.052 (3)	0.001 (3)	−0.013 (2)	−0.014 (3)
C4	0.037 (2)	0.055 (3)	0.040 (2)	−0.010 (2)	−0.016 (2)	−0.010 (2)
C5	0.038 (2)	0.032 (2)	0.0241 (19)	−0.0078 (18)	−0.0155 (17)	0.0004 (17)
C6	0.037 (2)	0.033 (2)	0.0236 (19)	−0.0105 (18)	−0.0107 (17)	−0.0037 (17)
C7	0.059 (3)	0.040 (2)	0.041 (2)	−0.020 (2)	−0.019 (2)	−0.011 (2)
C8	0.068 (3)	0.033 (2)	0.042 (2)	−0.005 (2)	−0.022 (2)	−0.014 (2)
C9	0.067 (3)	0.064 (3)	0.048 (3)	−0.022 (2)	−0.021 (2)	−0.017 (2)
C10	0.072 (3)	0.087 (4)	0.045 (3)	−0.011 (3)	−0.030 (3)	−0.026 (3)
C11	0.063 (3)	0.068 (3)	0.030 (2)	0.004 (3)	−0.020 (2)	−0.006 (2)
C12	0.046 (2)	0.042 (2)	0.032 (2)	−0.0029 (19)	−0.0107 (19)	−0.0053 (19)
C13	0.0270 (19)	0.032 (2)	0.0267 (19)	0.0024 (16)	−0.0052 (16)	−0.0075 (17)
C14	0.033 (2)	0.030 (2)	0.027 (2)	−0.0046 (17)	−0.0037 (17)	−0.0100 (17)
C15	0.058 (3)	0.039 (2)	0.042 (2)	−0.022 (2)	−0.009 (2)	−0.007 (2)
C16	0.063 (3)	0.047 (3)	0.051 (3)	−0.028 (2)	−0.008 (2)	−0.019 (2)
C17	0.042 (2)	0.029 (2)	0.028 (2)	−0.0002 (18)	−0.0095 (19)	−0.0121 (17)
C18	0.030 (2)	0.030 (2)	0.0287 (19)	−0.0084 (16)	−0.0068 (17)	−0.0146 (17)
C19	0.046 (2)	0.037 (2)	0.037 (2)	0.0019 (19)	−0.010 (2)	−0.0114 (19)
C20	0.048 (3)	0.056 (3)	0.045 (3)	0.006 (2)	0.000 (2)	−0.028 (2)
C21	0.062 (3)	0.059 (3)	0.027 (2)	−0.005 (2)	0.001 (2)	−0.017 (2)
C22	0.051 (3)	0.043 (2)	0.028 (2)	0.000 (2)	−0.010 (2)	−0.0078 (19)
C23	0.031 (2)	0.0283 (19)	0.030 (2)	−0.0057 (16)	−0.0089 (17)	−0.0122 (17)
C24	0.041 (2)	0.028 (2)	0.035 (2)	−0.0080 (18)	−0.006 (2)	−0.0080 (18)

### Geometric parameters (Å, °)

**Table:**

Cd1—N5	2.241 (3)	C4—H4	0.9300
Cd1—N2	2.304 (3)	C5—C6	1.456 (5)
Cd1—O1	2.306 (3)	C6—C7	1.394 (5)
Cd1—O5	2.306 (2)	C7—C8	1.360 (5)
Cd1—N1	2.355 (3)	C7—H7	0.9300
Cd1—N4	2.525 (3)	C8—H8	0.9300
O1—C17	1.263 (4)	C9—C10	1.378 (6)
O2—C17	1.246 (4)	C9—H9	0.9300
O3—C24	1.248 (4)	C10—C11	1.367 (6)
O4—C24	1.262 (4)	C10—H10	0.9300
O5—H5A	0.8500	C11—C12	1.372 (5)
O5—H5B	0.8500	C11—H11	0.9300
N1—C1	1.334 (5)	C12—C13	1.387 (5)
N1—C5	1.352 (4)	C12—H12	0.9300
N2—N3	1.336 (4)	C13—C14	1.461 (5)
N2—C6	1.340 (4)	C14—C15	1.389 (5)
N3—C8	1.336 (4)	C15—C16	1.363 (5)
N3—H3A	0.8600	C15—H15	0.9300
N4—C9	1.331 (5)	C16—H16	0.9300
N4—C13	1.345 (4)	C17—C18	1.513 (5)
N5—C14	1.336 (4)	C18—C19	1.385 (5)
N5—N6	1.343 (4)	C18—C23	1.395 (5)
N6—C16	1.329 (4)	C19—C20	1.377 (5)
N6—H6	0.8600	C19—H19	0.9300
C1—C2	1.369 (5)	C20—C21	1.367 (5)
C1—H1	0.9300	C20—H20	0.9300
C2—C3	1.373 (6)	C21—C22	1.372 (5)
C2—H2	0.9300	C21—H21	0.9300
C3—C4	1.360 (6)	C22—C23	1.394 (5)
C3—H3	0.9300	C22—H22	0.9300
C4—C5	1.389 (5)	C23—C24	1.500 (5)
			
N5—Cd1—N2	146.39 (10)	C8—C7—C6	105.2 (3)
N5—Cd1—O1	89.21 (9)	C8—C7—H7	127.4
N2—Cd1—O1	123.55 (9)	C6—C7—H7	127.4
N5—Cd1—O5	91.79 (10)	N3—C8—C7	107.9 (4)
N2—Cd1—O5	86.61 (10)	N3—C8—H8	126.1
O1—Cd1—O5	81.39 (9)	C7—C8—H8	126.1
N5—Cd1—N1	128.46 (10)	N4—C9—C10	123.9 (4)
N2—Cd1—N1	70.85 (11)	N4—C9—H9	118.1
O1—Cd1—N1	79.65 (10)	C10—C9—H9	118.1
O5—Cd1—N1	134.70 (10)	C11—C10—C9	117.8 (4)
N5—Cd1—N4	67.54 (10)	C11—C10—H10	121.1
N2—Cd1—N4	86.92 (10)	C9—C10—H10	121.1
O1—Cd1—N4	141.67 (10)	C10—C11—C12	120.0 (4)
O5—Cd1—N4	127.21 (10)	C10—C11—H11	120.0
N1—Cd1—N4	91.24 (10)	C12—C11—H11	120.0
C17—O1—Cd1	131.2 (2)	C11—C12—C13	118.7 (4)
Cd1—O5—H5A	130.4	C11—C12—H12	120.7
Cd1—O5—H5B	107.9	C13—C12—H12	120.7
H5A—O5—H5B	116.4	N4—C13—C12	122.0 (3)
C1—N1—C5	117.7 (3)	N4—C13—C14	115.6 (3)
C1—N1—Cd1	125.5 (3)	C12—C13—C14	122.4 (3)
C5—N1—Cd1	116.6 (2)	N5—C14—C15	110.1 (3)
N3—N2—C6	105.9 (3)	N5—C14—C13	117.4 (3)
N3—N2—Cd1	136.5 (2)	C15—C14—C13	132.5 (3)
C6—N2—Cd1	117.0 (2)	C16—C15—C14	105.1 (3)
C8—N3—N2	111.2 (3)	C16—C15—H15	127.4
C8—N3—H3A	124.4	C14—C15—H15	127.4
N2—N3—H3A	124.4	N6—C16—C15	108.0 (3)
C9—N4—C13	117.6 (3)	N6—C16—H16	126.0
C9—N4—Cd1	127.5 (3)	C15—C16—H16	126.0
C13—N4—Cd1	114.9 (2)	O2—C17—O1	126.4 (3)
C14—N5—N6	105.8 (3)	O2—C17—C18	117.4 (3)
C14—N5—Cd1	124.4 (2)	O1—C17—C18	116.1 (3)
N6—N5—Cd1	129.7 (2)	C19—C18—C23	119.4 (3)
C16—N6—N5	111.1 (3)	C19—C18—C17	118.2 (3)
C16—N6—H6	124.5	C23—C18—C17	122.3 (3)
N5—N6—H6	124.5	C20—C19—C18	121.0 (4)
N1—C1—C2	123.7 (4)	C20—C19—H19	119.5
N1—C1—H1	118.2	C18—C19—H19	119.5
C2—C1—H1	118.2	C21—C20—C19	119.9 (4)
C1—C2—C3	118.1 (4)	C21—C20—H20	120.1
C1—C2—H2	121.0	C19—C20—H20	120.1
C3—C2—H2	121.0	C20—C21—C22	119.9 (4)
C4—C3—C2	119.9 (4)	C20—C21—H21	120.0
C4—C3—H3	120.1	C22—C21—H21	120.0
C2—C3—H3	120.1	C21—C22—C23	121.4 (4)
C3—C4—C5	119.2 (4)	C21—C22—H22	119.3
C3—C4—H4	120.4	C23—C22—H22	119.3
C5—C4—H4	120.4	C22—C23—C18	118.3 (3)
N1—C5—C4	121.4 (4)	C22—C23—C24	119.2 (3)
N1—C5—C6	115.9 (3)	C18—C23—C24	122.5 (3)
C4—C5—C6	122.6 (3)	O3—C24—O4	124.5 (4)
N2—C6—C7	109.8 (3)	O3—C24—C23	118.6 (3)
N2—C6—C5	119.2 (3)	O4—C24—C23	116.8 (3)
C7—C6—C5	131.0 (3)		
			
N5—Cd1—O1—C17	−56.3 (3)	C3—C4—C5—N1	0.5 (5)
N2—Cd1—O1—C17	115.7 (3)	C3—C4—C5—C6	179.5 (3)
O5—Cd1—O1—C17	35.6 (3)	N3—N2—C6—C7	0.7 (4)
N1—Cd1—O1—C17	174.3 (3)	Cd1—N2—C6—C7	−171.7 (2)
N4—Cd1—O1—C17	−107.0 (3)	N3—N2—C6—C5	178.9 (3)
N5—Cd1—N1—C1	−31.9 (3)	Cd1—N2—C6—C5	6.6 (4)
N2—Cd1—N1—C1	179.9 (3)	N1—C5—C6—N2	−2.3 (5)
O1—Cd1—N1—C1	48.7 (3)	C4—C5—C6—N2	178.6 (3)
O5—Cd1—N1—C1	115.5 (3)	N1—C5—C6—C7	175.5 (4)
N4—Cd1—N1—C1	−93.8 (3)	C4—C5—C6—C7	−3.6 (6)
N5—Cd1—N1—C5	152.8 (2)	N2—C6—C7—C8	−0.6 (4)
N2—Cd1—N1—C5	4.6 (2)	C5—C6—C7—C8	−178.6 (4)
O1—Cd1—N1—C5	−126.5 (2)	N2—N3—C8—C7	0.1 (4)
O5—Cd1—N1—C5	−59.8 (3)	C6—C7—C8—N3	0.3 (4)
N4—Cd1—N1—C5	90.9 (2)	C13—N4—C9—C10	0.0 (7)
N5—Cd1—N2—N3	53.0 (4)	Cd1—N4—C9—C10	−177.2 (3)
O1—Cd1—N2—N3	−112.4 (3)	N4—C9—C10—C11	0.3 (7)
O5—Cd1—N2—N3	−35.1 (3)	C9—C10—C11—C12	−0.3 (7)
N1—Cd1—N2—N3	−175.1 (3)	C10—C11—C12—C13	0.0 (6)
N4—Cd1—N2—N3	92.5 (3)	C9—N4—C13—C12	−0.3 (5)
N5—Cd1—N2—C6	−137.6 (2)	Cd1—N4—C13—C12	177.2 (3)
O1—Cd1—N2—C6	56.9 (3)	C9—N4—C13—C14	−179.6 (3)
O5—Cd1—N2—C6	134.2 (2)	Cd1—N4—C13—C14	−2.1 (4)
N1—Cd1—N2—C6	−5.8 (2)	C11—C12—C13—N4	0.3 (6)
N4—Cd1—N2—C6	−98.2 (2)	C11—C12—C13—C14	179.5 (4)
C6—N2—N3—C8	−0.5 (4)	N6—N5—C14—C15	−0.7 (4)
Cd1—N2—N3—C8	169.6 (3)	Cd1—N5—C14—C15	175.8 (2)
N5—Cd1—N4—C9	177.2 (4)	N6—N5—C14—C13	178.7 (3)
N2—Cd1—N4—C9	19.5 (3)	Cd1—N5—C14—C13	−4.8 (4)
O1—Cd1—N4—C9	−126.0 (3)	N4—C13—C14—N5	4.3 (5)
O5—Cd1—N4—C9	102.9 (3)	C12—C13—C14—N5	−175.0 (3)
N1—Cd1—N4—C9	−51.2 (4)	N4—C13—C14—C15	−176.5 (4)
N5—Cd1—N4—C13	−0.1 (2)	C12—C13—C14—C15	4.3 (6)
N2—Cd1—N4—C13	−157.7 (3)	N5—C14—C15—C16	0.0 (5)
O1—Cd1—N4—C13	56.8 (3)	C13—C14—C15—C16	−179.2 (4)
O5—Cd1—N4—C13	−74.3 (3)	N5—N6—C16—C15	−1.1 (5)
N1—Cd1—N4—C13	131.5 (2)	C14—C15—C16—N6	0.6 (5)
N2—Cd1—N5—C14	46.0 (4)	Cd1—O1—C17—O2	30.6 (6)
O1—Cd1—N5—C14	−146.1 (3)	Cd1—O1—C17—C18	−152.7 (2)
O5—Cd1—N5—C14	132.5 (3)	O2—C17—C18—C19	116.8 (4)
N1—Cd1—N5—C14	−70.0 (3)	O1—C17—C18—C19	−60.2 (4)
N4—Cd1—N5—C14	2.6 (3)	O2—C17—C18—C23	−58.5 (5)
N2—Cd1—N5—N6	−138.4 (3)	O1—C17—C18—C23	124.5 (4)
O1—Cd1—N5—N6	29.5 (3)	C23—C18—C19—C20	2.8 (5)
O5—Cd1—N5—N6	−51.8 (3)	C17—C18—C19—C20	−172.6 (4)
N1—Cd1—N5—N6	105.6 (3)	C18—C19—C20—C21	−0.4 (6)
N4—Cd1—N5—N6	178.2 (3)	C19—C20—C21—C22	−0.9 (7)
C14—N5—N6—C16	1.1 (4)	C20—C21—C22—C23	−0.2 (6)
Cd1—N5—N6—C16	−175.2 (3)	C21—C22—C23—C18	2.6 (5)
C5—N1—C1—C2	−1.5 (6)	C21—C22—C23—C24	−175.2 (3)
Cd1—N1—C1—C2	−176.7 (3)	C19—C18—C23—C22	−3.8 (5)
N1—C1—C2—C3	1.7 (6)	C17—C18—C23—C22	171.4 (3)
C1—C2—C3—C4	−0.7 (7)	C19—C18—C23—C24	173.9 (3)
C2—C3—C4—C5	−0.3 (6)	C17—C18—C23—C24	−10.8 (5)
C1—N1—C5—C4	0.4 (5)	C22—C23—C24—O3	149.6 (3)
Cd1—N1—C5—C4	176.0 (3)	C18—C23—C24—O3	−28.2 (5)
C1—N1—C5—C6	−178.7 (3)	C22—C23—C24—O4	−27.2 (5)
Cd1—N1—C5—C6	−3.1 (4)	C18—C23—C24—O4	155.1 (3)

### Hydrogen-bond geometry (Å, °)

**Table:**

*D*—H···*A*	*D*—H	H···*A*	*D*···*A*	*D*—H···*A*
O5—H5A···O3^i^	0.85	1.84	2.672 (4)	168
O5—H5B···O3	0.85	2.07	2.892 (4)	164
N3—H3A···O4^i^	0.86	1.79	2.646 (4)	176
N6—H6···O2	0.86	1.91	2.700 (4)	152

Symmetry codes: (i) −*x*+1, −*y*, −*z*+1.

## References

[bb1] Borrows, A. D., Chan, C. M., Chowdhry, M. M., Mcgrady, J. E. & Mingos, D. M. P. (1995). *Chem. Soc. Rev.* **24**, 329–340.

[bb2] Bruker (2007). *APEX2* and *SAINT* Bruker AXS Inc., Madison, Wisconsin, USA.

[bb3] Cheng, J.-W., Zhang, J., Zheng, S.-T., Zhang, M.-B. & Yang, G.-Y. (2006). *Angew. Chem. Int. Ed.* **45**, 73–77.

[bb4] Hu, T.-L., Zou, R.-Q., Li, J.-R. & Bu, X.-H. (2008). *Dalton Trans.* pp. 1302–1311. 10.1039/b716398c 18305842

[bb5] Hunter, C. A. (1994). *Chem. Soc. Rev.* **23**, 101–110.

[bb6] Sheldrick, G. M. (1996). *SADABS* University of Göttingen, Germany.

[bb7] Sheldrick, G. M. (2008). *Acta Cryst.* A**64**, 112–122. 10.1107/S0108767307043930 18156677

[bb8] Wan, Y.-H., Zhang, L.-P., Jin, L.-P., Gao, S. & Lu, S.-Z. (2003). *Inorg. Chem.* **42**, 4985–4994. 10.1021/ic034258c 12895124

